# Solar potential assessment using machine learning and climate change projections for long-term energy planning

**DOI:** 10.1038/s41598-025-23661-0

**Published:** 2025-11-14

**Authors:** B. Nishant Sree Reddy, Kumar Gautam, Nikhil Pachauri

**Affiliations:** 1 Department of Mechatronics, Manipal Institute of Technology, Manipal Academy of Higher Education, Manipal, 576104 Karnataka India; 2 Quantum Computing Department, Quantum Research and Centre of Excellence, New Delhi, Delhi India

**Keywords:** Solar potential, Machine learning, XGBoost, LSTM, Tilt angle, Electrical and electronic engineering, Energy infrastructure

## Abstract

This work proposes a novel method for evaluating solar potential, essential for the development, installation, and operation of solar power systems. The approach forecasts solar energy potential for specific sites by utilizing integrated geospatial, meteorological, and infrastructural multidimensional data. A new application has been released to assess the solar capacity globally. The study evaluated various machine learning methods, ultimately selecting an XGBoost model for training on historical sun irradiance and meteorological data spanning from 1980 to 2015. This model demonstrates significant promise for handling complicated nonlinear interactions and simulating temporal weather patterns affecting solar irradiance. Preliminary results indicate a strong capacity for worldwide predictions on the potential of solar energy, utilizing simulated weather data from 2015 to 2099. The application delivers precise solar power estimates and financial viability, enabling rapid and effortless site assessments from any location within minutes. The results demonstrate that the XGBoost model outperforms other ML algorithms, by achieving lower values of RMSE = 0.97 kWh/m² and MAE = 0.76 kWh/m², respectively, for solar energy potential. Furthermore, to evaluate the impact of the proposed methodology, three case studies were conducted in Mindanao (Philippines), Gobi-Altai (Mongolia), and the Peloponnese (Greece). The results demonstrate the efficacy of the proposed method in long-term solar energy planning.

## Introduction

**Fig. 1:**
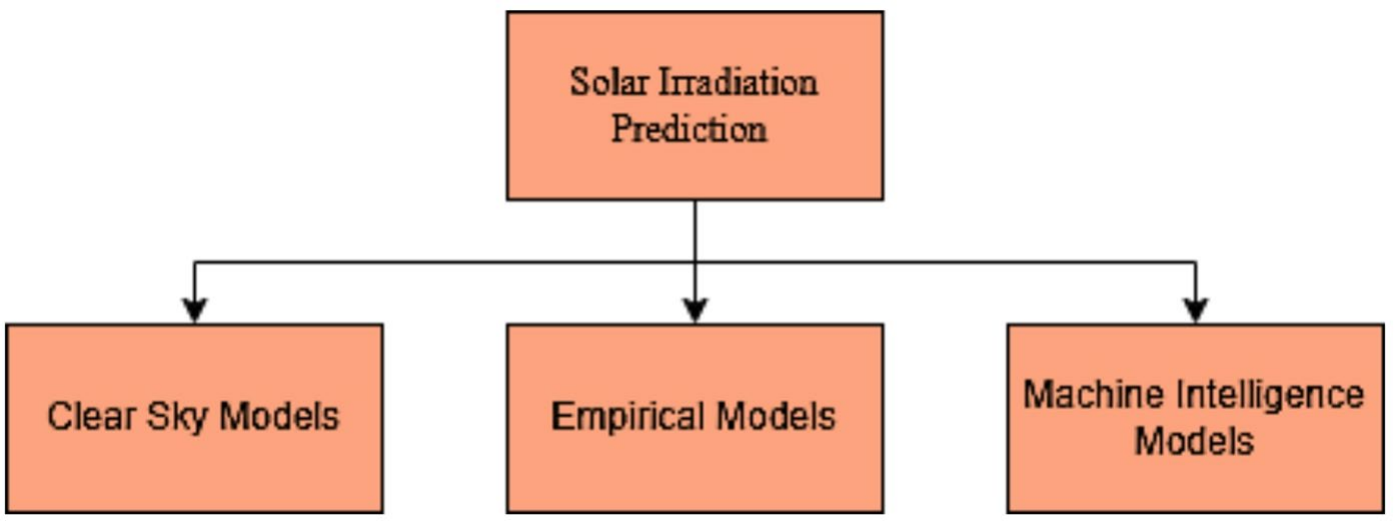
Different models for solar irradiation prediction.

Human emissions of greenhouse gases are the primary driver of significant alterations in Earth’s climate. Any approach to mitigate the risks associated with climate change must focus primarily on diminishing greenhouse gas emissions. Natural gas and other traditional fossil fuels are essential for the energy security of the decarbonisation process (Yang et al., 2022^1^). The Intergovernmental Panel on Climate Change (IPCC)^33^ reports indicate an urgent necessity for swift and extensive modifications in energy systems to restrict global warming to 1.5 °C above pre-industrial levels, achieved by transitioning from non-renewable to renewable energy sources (Intergovernmental Panel on Climate Change (IPCC), 2023). However, transitioning from fossil fuels to renewable energy is essential to ensure a comprehensive economic transformation aimed at mitigating climate change. The advancement of renewable energy sources, including wave, tidal, wind, and solar power, will substantially mitigate the progression of climate change. The primary reduction in carbon emissions is expected to derive from renewable energy sources, chiefly solar and wind energy (Yang et al., 2022^1^). Solar energy refers to renewable energy produced from the sun. It is utilised in several applications, including photovoltaic panels, which convert solar radiation into electrical energy for use as a power source. Solar energy can be utilised to heat water and produce steam to drive turbines that generate electricity. Solar energy is classified as a renewable resource, as the sun represents an inexhaustible power supply from the perspective of human timescales. Solar energy is regarded as a clean energy source since it generates electricity without emitting pollutants. Solar energy, characterised by its plentiful availability and declining technological expenses, emerges as a viable strategy for mitigating carbon emissions (Ledmaoui et al., 2023^2^). Advancements in climate modelling are yielding potential future atmospheric conditions, serving as a significant resource for long-term solar energy planning. Models utilised in the Coupled Model Intercomparison Project Phase 6 (CMIP6) offer forecasts of essential solar radiation variables. The existing solar energy planning techniques, which depend on historical data or short-term forecasts, may neglect long-term climate patterns that could affect solar project efficacy. The historical climate data and future climate model data are utilized to develop an application that forecasts solar potential over an extended period. Typically, photovoltaic systems utilise semiconductor materials, such as silicon, to generate electricity by energising electrons with supplementary energy. These devices operate on the idea that electrons are elevated from a lower energy state to a higher energy state through the infusion of energy from sunshine. This activation will subsequently generate many vacancies and release electrons in the semiconductor, so producing electricity. Monocrystalline silicon, microcrystalline silicon, cadmium telluride, polycrystalline silicon, and copper indium diselenide are the prevalent semiconductors utilised in solar systems. The selection of these materials is determined by multiple factors (Green, 2002^3^; Razykov et al., 2011^4^). Solar irradiance refers to the total quantity of solar energy incident on the Earth’s surface over a specified duration, measured in watts per square meter (W/m²). The predominant categories of solar irradiance include Diffuse Horizontal Irradiation (DHI), which refers to irradiance emanating from various directions rather than directly from the sun; Global Horizontal Irradiation (GHI), representing the cumulative direct and indirect energy absorbed by a horizontal surface per unit area; and Direct Normal Irradiation (DNI), which denotes the most direct solar radiation at a specific location (Sehrawat et al., 2023^5^). The forecast of solar irradiation can be distinctly categorised into three primary ways. The initial technique models are capable of predictions only in clear sky conditions and exhibit considerable complexity. These models account for the interaction of solar radiation with Earth’s atmosphere through absorption by water vapour, the ozone layer, and aerosols, as well as by Rayleigh scattering. The second method lacks great precision and employs averaged data. These models utilise the empirical correlations between the sunshine ratio (extraterrestrial irradiation) and the clearness index (horizontal global solar irradiation). Numerous models are stochastic in nature and are utilised across different temporal scales. Recent methodologies utilise various machine intelligence algorithms to analyse data for predicting irradiation based on weather information (El-Amarty et al., 2023^6^; Huang et al., 2021^7^). Figure 1 shows the types of solar irradiance predictive models.

### Related work

The prediction of solar energy under uncertain conditions is important as climate change introduces non-stationary patterns in atmospheric conditions, making historical averages increasingly unreliable for long-term planning. Solar investments have 25-year lifespans, requiring robust predictions under various climate scenarios. Additionally, grid integration and energy storage planning depend critically on understanding future variability in solar resources at the policy level; setting renewable energy targets requires uncertainty-quantified projections to ensure feasibility. As climate change becomes increasingly evident and accelerated, a swift switch to renewable energy sources is imperative. The Intergovernmental Panel on Climate Change (IPCC) has emphasised the urgent necessity for substantial and extensive reforms in global energy systems to restrict warming to 1.5 °C above pre-industrial levels (IPCC, 2023). In this context, solar energy is considered a fundamental element of decarbonisation plans, necessitating sophisticated approaches for precise assessment of solar potential across extensive geographical areas and prolonged temporal scales. This publication synthesises cutting-edge research on solar potential prediction, encompassing novel approaches, technical breakthroughs, and the integration of climate change concerns. The development of solar potential assessment methods reflects the growing sophistication of technological capabilities and analytical procedures. Early approaches were predominantly based on clear sky models and empirical relationships between the sunshine ratio and the clearness index, while the most recent approaches are much more advanced. El-Amarty et al. 2023^6^ give an extensive review of this evolutionary process, showing how the method moved from simple empirical models to advanced artificial neural networks and computational procedures. The changes were basically motivated by our knowledge that atmospheric and meteorological processes are non-linear and connected, so that expert solutions are required for estimating sun irradiance. There was a tremendous improvement in solar potential prediction accuracy due to the existence of ML-based approaches. Huang et al. 2021^7^, showed improved solar radiation forecasting based on different machine learning models, especially extreme climatic events. This change in methodology reflects an increased understanding of the nonlinear characteristics of multivariate dependencies of meteorological variables and solar radiation and allows a more comprehensive and statistically significant forecasting than that from traditional statistical cross-correlation. Taking climate change impacts into account in solar potential assessment is an important improvement in the framework of the long-term vision process. The study by Yang et al. 2022^1^ on the impact of climate change on wind and solar power generation in Europe highlights the importance of comprehensive impact assessments accounting for climate uncertainty. This requires adaptive evaluation methodologies, as their results point to the possibility of the significance of changes in solar resource’s availability and reliability characteristics. Their study highlights the importance of evaluating how changes in climate variation may affect both the supply and predictability of solar resources, with a need for flexible assessment techniques. The Latest generation of climate-informed solar potential assessments is from the Coupled Model Intercomparison Project Phase 6 (CMIP6). Eyring et al. (2016)^8^ describe the enhanced capacity of CMIP6 due to better representation of physical processes, higher resolution, and an expanded range of future simulations. O’Neill et al. (2016)^9^ significantly expand the capability of these complex climate models to support such a precise, long-term solar energy planning through alternative future projections that represent different socioeconomic futures. This integration of climate modeling with solar potential assessment represents a key step toward the ability of the field to account for future atmospheric conditions. In the work of Chen and Guestrin (2016)^10^, the XGBoost model shows very high accuracy for regression (R² = 0.9287) and exhibits very promising error metrics compared to standard methods. XGBoost has high performance mainly because of its robust algorithmic characteristics, i.e., regularisation and high-order non-linearity structure, which are reflected in the model. A significant literature addresses the performance of different machine-learning methodologies for different spatial and temporal scales. Voyant et al. (2017)^11^ prove that ensemble approaches outperform single-model methods for predicting solar radiation. Together, these results highlight the necessity of advanced machine learning techniques to improve solar potential predictions. A study of the optimization of solar panel orientation and tilt angle reveals important findings on the maximization of captured energy. Modarresi and Hosseinnia (2023)^12^ developed sophisticated models to find the perfect daily tilt angle in various locations all around the world, as an accurate orientation can seriously lead to increasing the energy production. Le Roux (2016)^13^ has also provided an experiment on the optimization of tilt and azimuth for solar collection efficiency in South Africa, which complements similar theoretical models. The orientation of panels and local conditions have been identified as a key consideration. The finding of Kadhim et al., (2023)^14^ accentuates that it is crucial to tailor the optimisation process according to the existing solar radiation behaviour in the Baghdad region. Together, these studies emphasize the importance of considering geographical as well as temporal factors when optimizing solar panel layout. Modern economic analysis of solar energy projects is based on more sophisticated techniques. Off-Grid Solar Photovoltaic Systems in Sub-Saharan Africa: Economic Factors Beyond Solar Capability Only. Baurzhan and Jenkins (2016) 31 also focus on the importance of the installation cost, operation, and maintenance costs, and local economic conditions affecting qualifying decisions. Technical-economic analyses have developed to a very high standard. For example, Kang and Rohatgi 2016^15^ was a literature review of quantitative research related to the LCOE for commercial-scale PVT systems. Their research shows that the direct as well as indirect costs, along with the direct and indirect benefits, need to be considered when judging the feasibility of a project. Acknowledgment of the Need for Solar Potential Evaluation. The need for solar potential evaluation, including both its environmental and social facets, has generated interest. One of the breakthrough studies in the societal acceptance of renewable energy innovations, Wüstenhagen et al. (2007)^16^ showed that the social side is crucial for the success of such projects. This work highlights the tremendous impact that social acceptance has on project viability, particularly in locations that possess significant technical expertise. Today, environmental considerations have become a main part of solar knowledge. Provide a firm basis for understanding the broader ecological implications of solar development. This research highlights the importance of including direct and indirect environmental impacts in project design and evaluation. The rapid development of photovoltaic technology changes the approaches used to determine the solar potential in each area. In this sense, the introduction of recent studies on photovoltaic principles meets at once bottlenecks and prospects in the field of solar energy conversion (Green, 2002^3^). Razykov et al. (2011)^4^. A comprehensive review of solar PV power reveals the continued manifestation of progress in technology on solar energy potential assessment and calculation. The development of solar cell metrology and the design of solar cells have necessitated several modifications in the approach to the evaluation. Recent developments in bifacial modules, trackers, and storage technologies have expanded the available options for capturing and using solar energy. Such technological progressions demand increasingly sophisticated test equipment that can support new features and topologies. The integration of solar resource assessment with grid layout has become crucial. Finally, the analysis by Sepulveda et al. (2018)^17^ on the role of low-carbon power resources for firms emphasizes the need to consider grid integration issues when assessing solar capabilities. The research shows that grid stability, transmission capacity, and capabilities for energy storage are key factors that determine how effectively we can use the solar potential. The tandem of solar potential and energy storage requirement is emerging to be a crucial issue. The study by Heard et al. (2017)^18^ demonstrates the need for storage when assessing solar potential in a renewable system. It demonstrates that due to the variability in the availability of solar resources, the storage requirement needs careful assessment. Many challenges as well as research opportunities for solar potential evaluation were identified. The need for improved coupling of climate change projections with the methods for determining solar potential remains a significant challenge. The research of (Eyring et al., 2019^19^) on the evaluation of climate models emphasizes the need for the continuation of improvement and upgrade of climate projection products to serve solar energy planning. The development of more sophisticated machine learning techniques and their combination with physics-based models opens a promising direction for future research. A hybrid ML algorithm based on XGBoost and GRU is proposed by Yaojian Xu et al.., (2024)^20^, for the long-term prediction of solar energy. The result suggests that the proposed algorithm reduces the MSE and MAE by 28% and 17.4% compared to other algorithms. Erbay (2025)^21^ proposed an XGBoost algorithm for solar forecasting and green hydrogen cost mapping in Türkiye. The simulation results revealed that XGBoost attains R^2^ value equal to 0.988 compared to other algorithms. A detailed comparative analysis of different ML algorithms combined with XGBoost is conducted by Nam et al. (2025)^22^ for short-term solar power forecasting. It can be observed from the results amalgamation of XGBoost with different algorithms has improved the overall predictive performance for the ML algorithms. Zhu et al. (2025), ^23^, proposed a combination of LSTM and XGBoost ML algorithms for the short-term forecasting of the power for a photovoltaic power station. It is observed from the results that it improved the overall prediction accuracy compared to standalone LSTM or XGBoost.

### Motivation and novelty

Due to climate change and the heightened utilisation of non-renewable energy sources, a global initiative advocates for a transition to 100% renewable energy. Solar energy is undoubtedly one of the most abundant and accessible energy sources, playing a significant part in this transformation. Photovoltaic (PV) panel technology has emerged as the predominant technology because of its favourable cost-to-efficiency ratio, ease of production, and widespread applicability. Nonetheless, the application of light energy mostly occurs during the light-dependent reactions of photosynthesis, and the efficiency of physical power conversion is very sensitive to sunlight (irradiance) and energy conversion processes. Solar irradiance is a variable signal, fluctuating over time due to meteorological conditions, diurnal cycles, geographical location, and seasonal changes. Consequently, precise forecasting of solar irradiance is crucial for determining the ideal site for solar installations and for optimising the power output of such systems^34,35^. Previous studies on solar prediction faced several challenges that are addressed in this work. Many studies relied on limited climate integration, often using simple scaling factors instead of full climate model outputs. Computational constraints also posed a barrier, as Physics-based models were too computationally intensive for global applications. In addition, earlier methods struggled to quantify confidence bounds on long-term predictions. Furthermore, prior studies focused on either short-term (hourly/daily) or annual averages, missing seasonal shifts critical for grid planning. In this work, a maiden attempt has been made to design an ML-based framework approach for assessing solar potential, which is crucial for the development, installation, and operation of solar energy systems. The method predicts solar energy potential for designated locations by employing combined geospatial, meteorological, and infrastructural multidimensional data. In addition to this, a comprehensive project feasible assessment report will be generated. It gives the information about the site characteristics, environmental impact metrics, solar resource metrics, and economic indicators for sustainable solar energy deployment.

## Dataset description

**Fig. 2:**
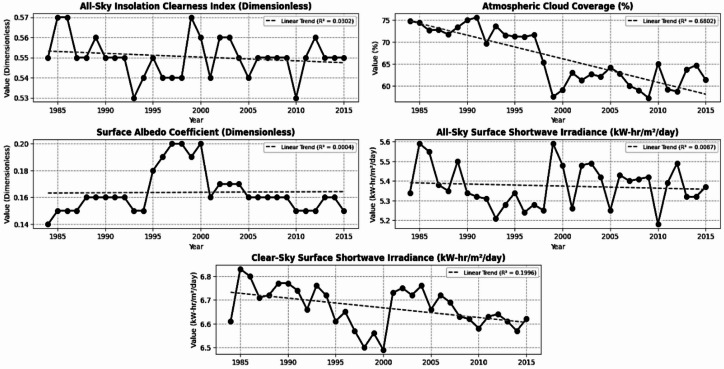
Historical weather plots.

For the application, raw historical weather data were first collected from the NASA  (https://power.larc.nasa.gov/)^30^  environmental database, a repository renowned for its extensive and meticulously curated meteorological records. The primary objective of the data acquisition phase is centred on extracting and reorganizing raw meteorological information with a focus on weather variables demonstrating direct or indirect correlational significance to global horizontal irradiance (GHI). Figure 2 shows the historical weather plots. A quality control method was implemented on the gathered raw database from the NASA POWER with a spatial resolution of 0.5° × 0.5° by eliminating physically implausible values, such as negative irradiance, identifying outliers through the 3-sigma rule, and corroborating with ground station data where accessible. Cubic spline interpolation is employed for short intervals of under three days, whereas seasonal decomposition is utilised for extended intervals, hence maintaining data integrity by restricting interpolation to less than 4% of the whole dataset. A min-max normalisation technique is employed for neural network models, but the original scale is preserved for tree-based models such as XGBoost and Random Forest. To enhance the prediction efficacy of the machine learning models, feature engineering is conducted by generating lagged variables (1-day, 3-day, 7-day), applying cyclical encoding for temporal features via sine and cosine transformations of the day/year, and computing moving averages to smooth trends. A time series of meteorological data with a daily average temporal resolution aggregated over 24 h is employed. The temporal coverage extends from historical data (January 1, 1980 - December 31, 2015, encompassing 13,149 days) to future forecasts (January 1, 2016 - December 31, 2099, totalling 30,681 days). It offers the global average at a spatial resolution of 0.5° (~ 55 km at the equator).

**Fig. 3:**
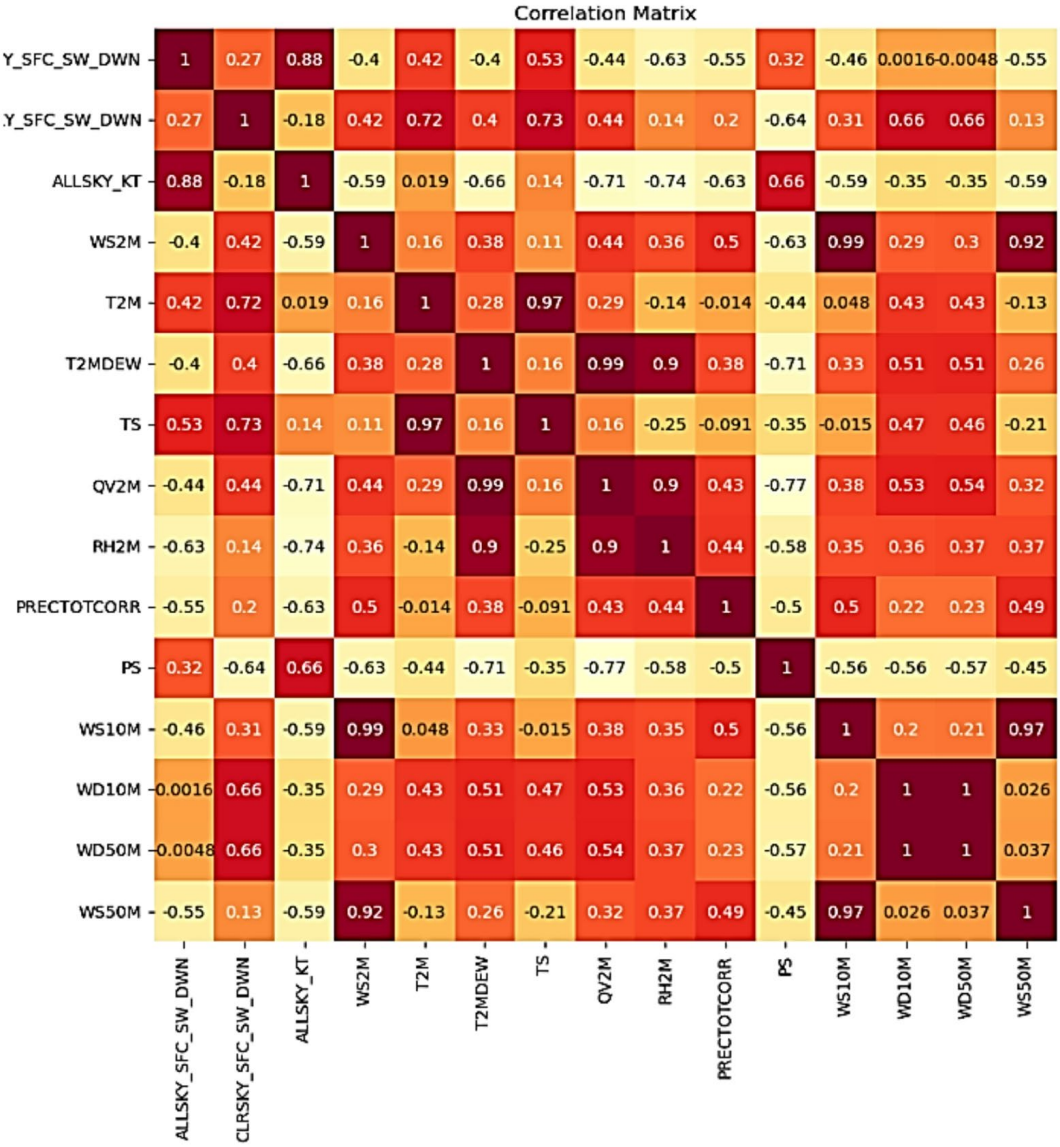
Pearson correlation matrix.

The weather features available are as follows cloud amount, shortwave downward irradiance (all sky and clear sky), temperature (earth skin, 2 m (m) height), dew/frost points (2 m height), specific humidity (2 m height), relative humidity (2 m height), precipitation, surface pressure, wind speed (10 m height (SMH), 50 m height (hub height reference)), wind direction (directional measurements at respective heights), all sky surface albedo, all sky isolation clearness index. Pearson correlation (Fig. 3) was used to build a matrix to find the best features that influence solar irradiance. The whole historical weather dataset is divided into a training dataset (70%), a validation dataset (15%), and a testing dataset (15%), respectively.

##  Materials and methodology

**Fig. 4:**
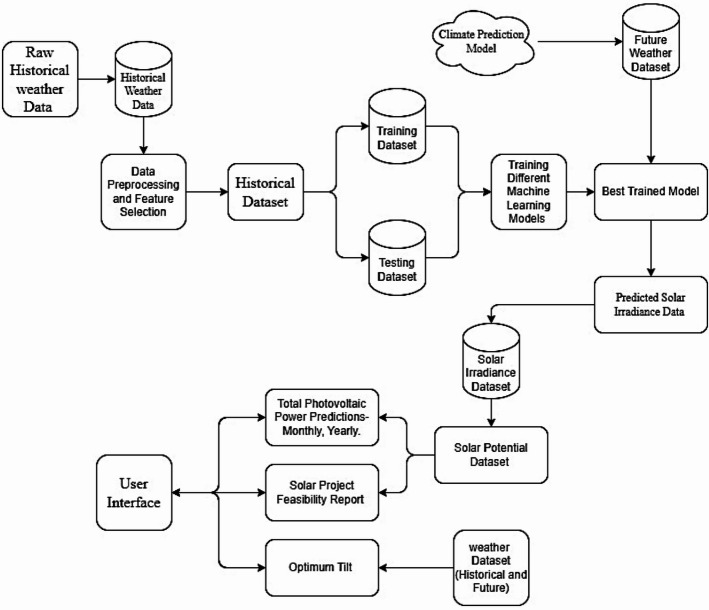
Flowchart for an overall methodology for the development of an application.

The methodology (Fig. 4) employs comprehensive, data-driven processes designed to generate precise and practical predictions of solar potential. It integrates NASA’s historical weather data (1980–2015) with CMIP6 model future climate scenarios (up to 2099) for comprehensive training and forecasting. The data undergoes extensive pre-processing, including normalisation, imputation, and feature selection, to render it more suitable for machine learning, particularly for GHI prediction. There are 15 input features that includes shortwave downward irradiance (clear sky), cloud amount, temperature (2 m), dew point (2 m), specific humidity (2 m), relative humidity (2 m), precipitation, surface pressure, wind speed (10 m), wind direction (10 m), surface albedo, clearness index, day of year (cyclical encoded), latitude (location feature), elevation and one output feature global horizontal irradiance (GHI). Multiple models were evaluated, and XGBoost emerged as the most effective model utilising Bayesian hyperparameter optimisation. A worldwide solar potential map is generated from the trained model, and a user interface is created in Tkinter for interactive location-specific analysis. The technology calculates the optimal panel tilt and orientation angle based on latitude and uses conventional energy conversion formulas to estimate the monthly and annual solar power output of a panel. It has a feasibility study function that considers economic, regulatory, and environmental criteria to assess the viability of a solar project. The system provides intuitive visualisations and reporting, enabling organisations to make decisions and act without requiring technical expertise.

Furthermore, ML models are compared to predict long-term solar potential, taking into consideration model accuracy, trustworthiness, and deployment capability. Four well-known performance measures are used for the evaluation: R² (Coefficient of determination)-Relative proportion of target variance explained by the model, RMSE (Root Mean Square Error)-dispersion of prediction errors, MAE (Mean Absolute Error)- average magnitude of error, not considering the direction, and RAE (Relative Absolute Error)- normalized prediction accuracy, also relatively to a base model. These measures have been selected due to their complementary properties, as they collectively represent a comprehensive assessment of the forecast performance of the two models. The model selection criteria are considered based on a well-defined tripartite structure that incorporates: (1) capability in capturing the nonlinear interactions between the meteorological variables, (2) computational efficiency suitable for large-scale, long-term usage, and (3) spatial, temporal robustness under the dynamic climatic data, as recommended in previous work (Wang et al., 2019^24^). This comparative analysis considers three types of machine learning paradigms: (i) linear regression variants, which act as interpretable benchmarks with established statistical properties, (ii) ensemble learning techniques, including Random Forest and XGBoost, which are renowned for their ability to capture complex nonlinear dependencies using a majority voting approach among a set of weak learners, and (iii) neural networks, which exploit multiple layers of feature transformation to learn patterns in high-dimensional data. A taxonomically based approach allows for a cross-paradigmatic comparison, enhancing methodological rigor and interpretative coherence (Voyant et al., 2017^11^). Within this framework, the best-performing algorithm for long-term solar potential prediction, both in terms of generalisation capability, accuracy, and computational scalability, will be selected.

### Linear regression-based models

- Multiple linear regression (MLR) constitutes a foundational statistical approach that models target variables as linear combinations of multiple predictors, offering methodological simplicity and interpretability. Within solar forecasting applications, MLR serves as an established baseline despite its inherent limitations in capturing nonlinear atmospheric interactions (Huang et al., 2013^25^).
- LASSO Regression extends the linear paradigm through L1 regularization, systematically performing feature selection by shrinking coefficients toward zero. This approach demonstrates enhanced robustness against multicollinearity frequently encountered in high-dimensional meteorological datasets (Shamshirband et al., 2015^26^).

### Tree-based ensemble methods

- Random Forest (RF) employs an ensemble of decorrelated decision trees trained on bootstrapped samples with random feature subsets. This methodological approach mitigates overfitting while capturing complex nonlinear relationships without distributional assumptions, a significant advantage for meteorological applications with heterogeneous atmospheric variables (Fan et al., 2018^27^).
- Gradient Boosting Machines (GBM) implement sequential ensemble construction, with each subsequent model concentrating on residual errors of preceding models. This iterative error-focusing approach enables progressive performance refinement, particularly beneficial for complex boundary conditions in solar radiation prediction (Voyant et al., 2017^11^).
- XGBoost represents an optimized implementation of gradient boosting that incorporates sophisticated regularization mechanisms and efficient tree construction algorithms (Chen & Guestrin, 2016^10^). The implementation features distributed computing capabilities and novel sparsity-aware algorithms for missing value handling—a common challenge in meteorological datasets.

### Neural network approaches

- Multilayer Perceptron (MLP) implements multiple interconnected neuronal layers with nonlinear activation functions, enabling complex function approximation without explicit feature engineering. This architecture offers flexible nonlinear modeling capabilities, particularly advantageous for capturing intricate meteorological interactions (Lima et al., 2016^28^).
- Long Short-Term Memory Networks (LSTM) incorporate specialized recurrent architectures designed to capture temporal dependencies in sequential data. This structural specialization makes LSTMs theoretically well-suited for solar irradiance prediction, where temporal weather patterns significantly influence outcomes (Qing & Niu, 2018^29^).

### Climate prediction models and future weather data

The Coupled Model Intercomparison Project Phase 6 (CMIP6) represents the latest generation of climate models and scenarios used to project future climate conditions. CMIP6 builds upon its predecessors by incorporating improved physical processes, higher spatial resolution, and a wider range of future scenarios, making it a valuable resource for long-term solar energy planning (Eyring et al., 2016^8^). CMIP6 includes contributions from over 40 modelling groups worldwide, offering an unprecedented ensemble of climate projections. The project features a matrix of simulations, including historical runs and future scenarios based on different Shared Socioeconomic Pathways (SSPs) and radiative forcing levels (O’Neill et al., 2016^9^). This diverse set of projections allows for a comprehensive assessment of potential future climate conditions relevant to solar energy production. For this application, future weather data was collected from CMIP6 models and scenarios and made into a future weather dataset.

### Solar power prediction-yearly, monthly

The power prediction in the selected coordinate does not contain site-specific obstacles such as shades, etc. The power prediction provides the theoretical maximum power generated in that coordinate or the site for a given solar panel area. It generates theoretical monthly and yearly power generation for the future years according to the predicted solar potential data. Equations (1) and (2) are used for the monthly and yearly solar power calculations.

1 $$\begin{aligned} Energy~Output~\left( {\frac{{Kwh}}{{Yearly}}} \right) = & ~Solar~Array~Area~\left( {M^{2} } \right) \times ~Conversion~Efficiency~ \\ & \times Solar~Radiation~for~The~Year\left( {Kwh/M^{2} /Year} \right) \\ \end{aligned}$$

2 $$\begin{aligned} Energy~Output~\left( {\frac{{Kwh}}{{Yearly}}} \right) = & ~Solar~Array~Area~\left( {M^{2} } \right) \times ~Conversion~Efficiency~ \\ & \times Solar~Radiation~for~The~Year\left( {Kwh/M^{2} /month} \right) \\ \end{aligned}$$

### Optimum orientation and tilt angle

**Fig. 5:**
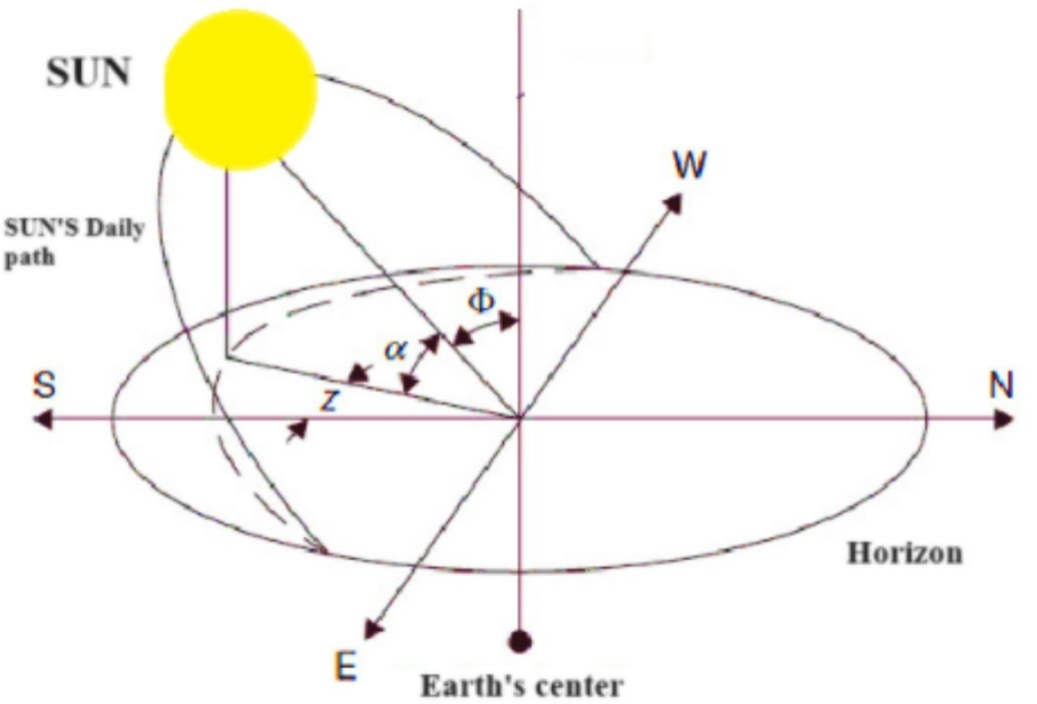
Sun’s daily path with respect to the Earth, where *α* is the solar altitude angle and Φ is the solar zenith angle.

Solar energy production depends on several factors, including the tilt angle and orientation, atmospheric conditions, and the sun’s position. Among all the factors, the tilt angle and orientation of the solar panels can be changed. Significant efforts have been made to identify the best orientation and optimal tilt angle for capturing the maximum solar radiation on the panel surface at different locations and various periods of time (Modarresi and Hosseinnia, 2023^12^). The optimum orientation for the solar panels in the northern hemisphere is facing true south, and in the southern hemisphere is facing true north (Kadhim et al., 2023^14^; Le Roux, 2016^13^). The daily optimum tilt angle of the surface from horizontal ($\:{\beta\:}_{op}$) is calculated by the formula

3 $$\:{\beta\:}_{op}\:=\:A\:+\:B\:\times\:\:cos(\omega\:n\:+\:\theta\:)$$

where DC value in southern hemisphere is A = 0.79φ − 1.47 and northern hemisphere is A = 0.83φ + 0.62, cosine function amplitude in southern hemisphere is B = − 0.04φ + 31.89 and northern hemisphere is B = 0.06φ + 31.29, θ is the phase which is constant with θ = 9.94, ω = 2πf, and f is the frequency of the optimum tilt angle, ϕ is the latitude of the location and If the average value of $\:{\beta\:}_{op}\:$is calculated over a year, the obtained value is equal to A. So, A can be considered as the optimum yearly tilt angle (Modarresi and Hosseinnia, 2023^12^). The application gets the latitude of the selected position from the map and substitutes it into Eq. (3) to get the daily optimum tilt angles, and then calculates the optimum yearly and seasonal tilt value for the user (Figure 5).

## Simulation results

**Fig. 6:**
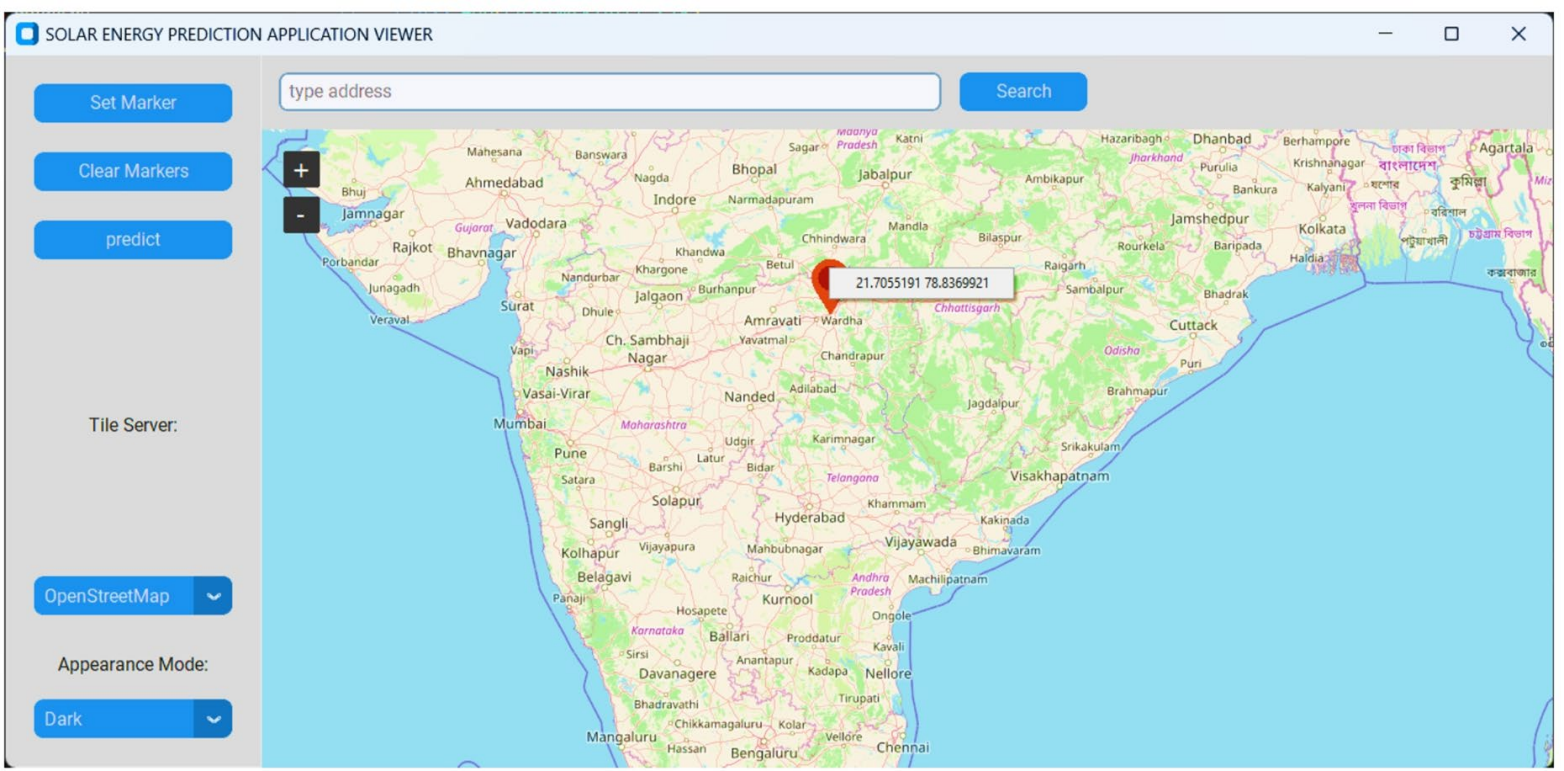
Location set marker in the interface. (https://customtkinter.tomschimansky.com/ and https://github.com/TomSchimansky/TkinterMapView/)

**Fig. 7:**
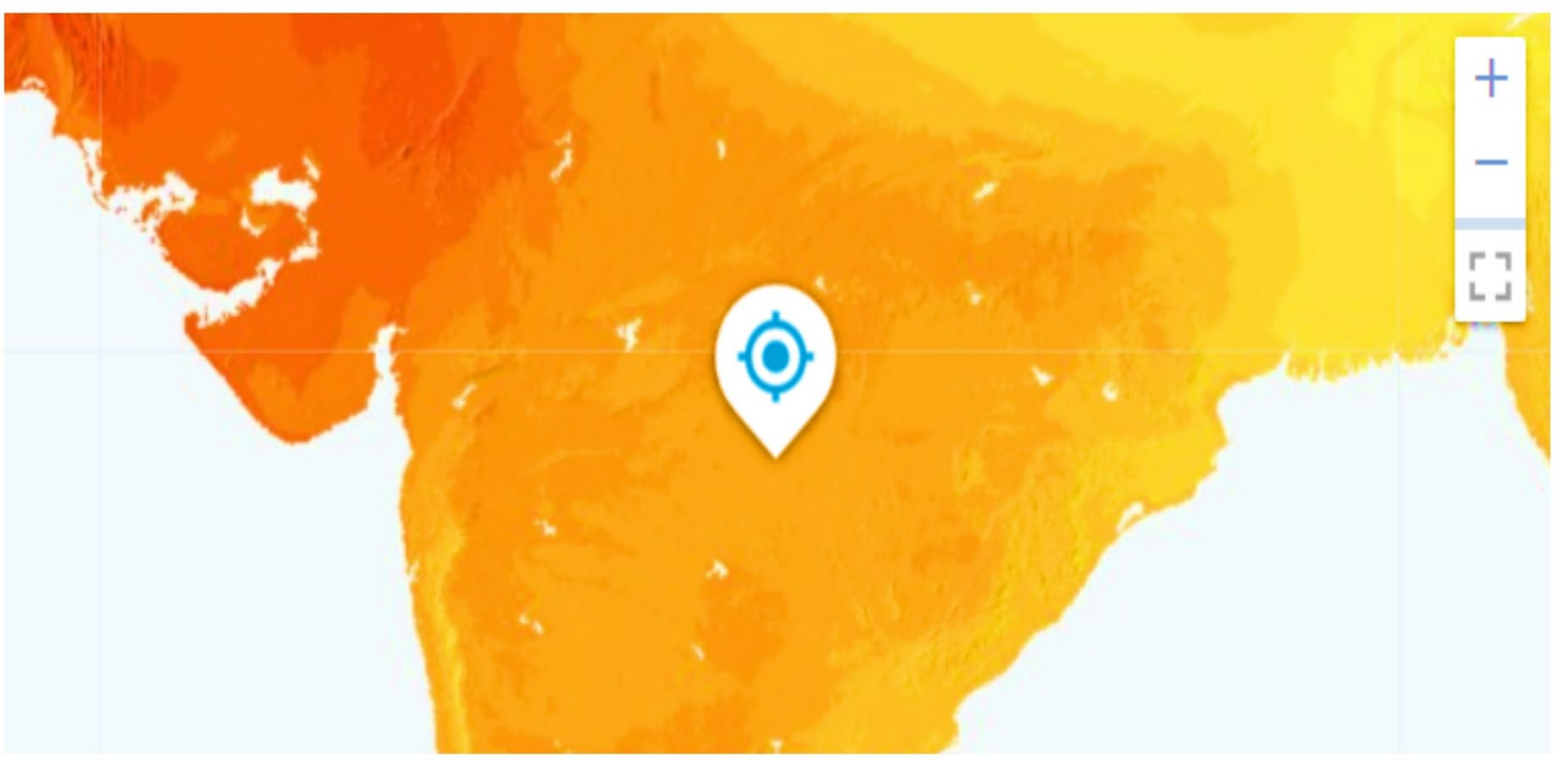
Masked solar potential map (https://matplotlib.org/).

The user interface is developed using Tkinter, the standard graphical user interface toolkit for Python. The web map contains multiple interrogation functionalities, allowing users to ‘click’ on a point to identify a location. Upon repositioning the marker to the preferred place, the user selects the “Predict” button to initiate the prediction. This retrieves solar potential data based on the chosen coordinates and then offers location-specific information, including estimated solar irradiance, optimal panel tilt, and energy yield calculations. The design is intended to be transparent to the end user, providing a natural experience while guaranteeing real-time feedback irrespective of the underlying processing complexity, as shown in Fig. 6. Furthermore, solar irradiance is calculated for each location on the world map using future meteorological data by creating scenarios (SSP1-2.6: sustainability scenario (1.5 °C warming), SSP2-4.5: middle road scenario (2.7 °C warming), SSP5-8.5: fossil-fuel development (4.4 °C warming)). Upon user selection of a coordinate (selected coordinates 21.42 N and 78.50 E), the system extracts solar potential data from the predictive map and displays it to the users as illustrated in Fig. 7. However, both the figure generated using Custom Tkinter (Modern GUI framework, Version-5.2.2) and Tkinter Map View (Interactive map widget, Version-1.29) software in Python. The Matplotlib library is used to generate a heat map (Fig. 7). In addition, diverse ML techniques have been employed to forecast solar potential and produce the solar feasibility report. A thorough comparative study was conducted across all implemented models, assessing performance across several indicators and differing geographical and meteorological variables. Table 1 shows the training evolution metrics for XGBoost.

**Table 1:** Training evolution metrics for XGBoost.

Epoch	Training RMSE	Validation RMSE	Testing RMSE	Training RAE	Validation RAE	Testing RAE
50	2.43	2.51	2.48	0.421	0.438	0.432
100	1.89	1.95	1.93	0.328	0.341	0.337
150	1.52	1.58	1.56	0.264	0.276	0.272
200	1.28	1.34	1.33	0.223	0.234	0.232
250	1.13	1.19	1.18	0.197	0.208	0.206
300	1.04	1.09	1.08	0.181	0.190	0.189
350	0.99	1.03	1.02	0.172	0.180	0.178
400	0.96	0.99	0.99	0.167	0.173	0.173
450	0.95	0.98	0.97	0.165	0.171	0.170
500	0.94	0.97	0.97	0.164	0.170	0.169

**Fig. 8:**
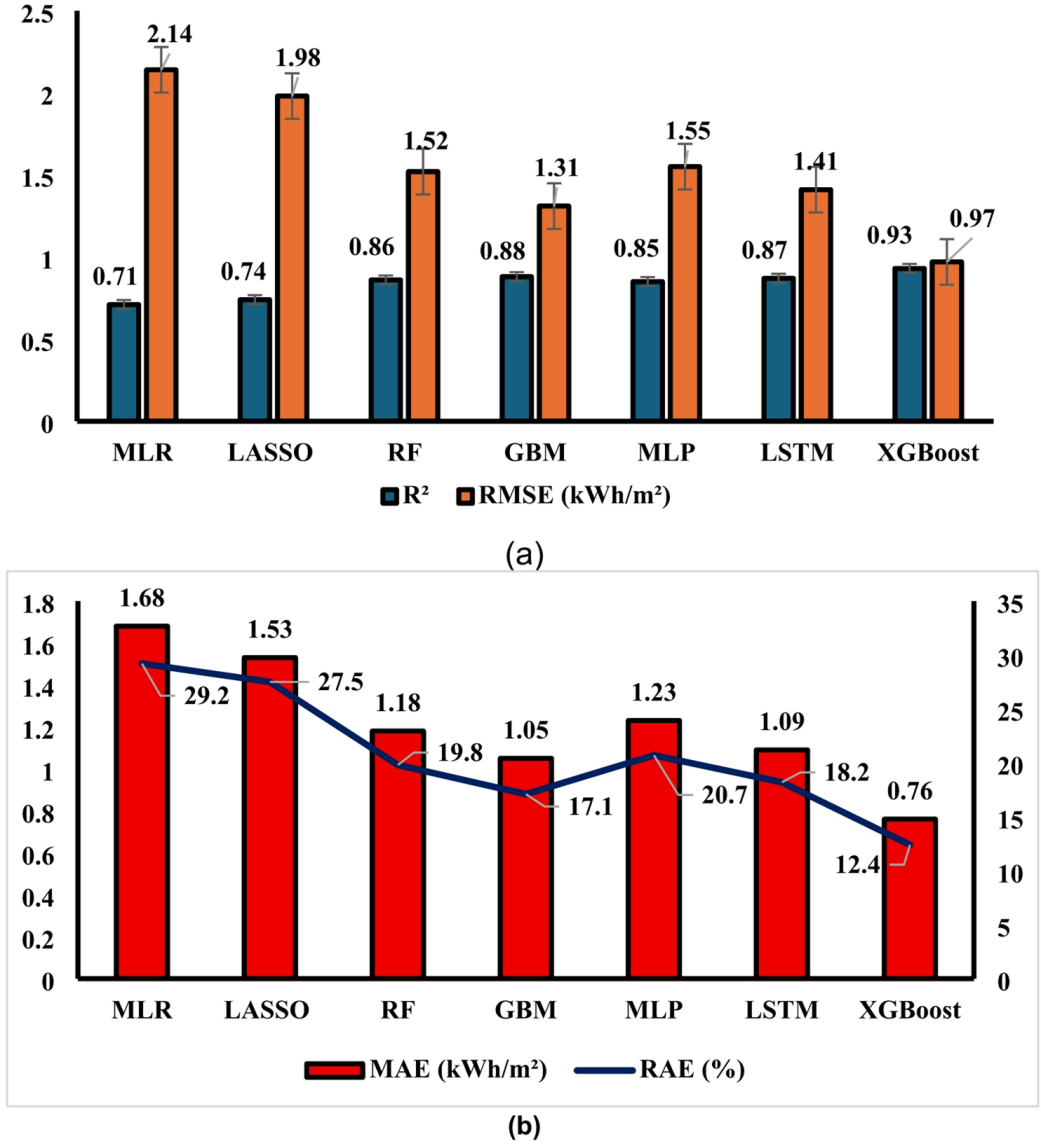
Comparative analysis of different ML algorithms.

It can be observed from Table 1 that early stopping analysis indicated an optimal termination point at epoch 450, where validation loss stopped improving. Significantly, there were no signs of overfitting, as both the training and validation losses stabilised, and test set performance remained consistent after epoch 400. The patterns of change are evident in the learning curves: initially (epochs 0–150), the RMSE declines rapidly from 2.43 to 1.52; subsequently, during epochs 150–400, the model converges gradually with a low RMSE reduction; and in the saturation phase (epochs 400–500), the RMSE improvement is negligible, indicating that the model has reached its capacity. The tight alignment of validation loss, test loss, and training loss reflects the model’s effective generalisation. Furthermore, the comparative analysis (Fig. 8a) of solar potential prediction using machine learning models indicated a notable enhancement after implementing various algorithmic approaches. LR had moderate predictive capabilities, evidenced by an R² value of 0.71 and an RMSE of 2.14 kWh/m², indicating its effectiveness in early-stage forecasting while highlighting deficiencies in high-accuracy predictions. In contrast, LASSO demonstrated reduced accuracy (R², 0.74; RMSE, 1.98 kWh/m²), showing marginal enhancement of LR overall, although exhibiting superior concordance in high-dimensional meteorological contexts. The little enhancements in performance underscore the limitations of linear models in depicting atmospheric interactions. The enhancement resulted from the transition to ensemble methods. Five hundred estimators of optimised depth-constrained RF achieved an R² of 0.86 and an RMSE of 1.52 kWh/m², demonstrating the RF’s potential capability to capture the entire impacts of cloud cover and atmospheric conditions on solar irradiance heterogeneity. The GBM implementation demonstrated further enhancements, achieving an R² of 0.88 and an RMSE of 1.31 kWh/m², particularly excelling under diverse climatic circumstances. The observed outcomes align with theoretical insights regarding successive ensemble benefits in intricate meteorological interactions. Compared to all tested models, XGBoost demonstrated exceptional results across most measures, achieving an R² of 0.93 and an RMSE of 0.97 kWh/m², indicating much higher predictive performance across various geographical locations and atmospheric conditions. This observation corroborates prior research regarding the algorithm’s efficacy in structured prediction challenges, including non-linear feature interactions. The neural methodologies yielded competitive, if not superior, outcomes. The Multi-Layer Perceptron (MLP) model 9 achieved an R² of 0.85 and an RMSE of 1.55 kWh/m², requiring extensive hyperparameter adjustment to mitigate overfitting related to the temporal characteristics of meteorological data. The LSTM demonstrates potential with R² = 0.87 and RMSE = 1.41 kWh/m², particularly in capturing seasonal and daily radiation patterns, while it remains a computational burden for practical application. It is also observed from Fig. 8b that XGBoost attains the lower values of MSE (kWh/m^2^) and RAE (%) compared to other algorithms. This study highlights the varying performances of machine learning models in predicting solar potential, underscoring the importance of selecting an appropriate model for a certain forecasting task and feature condition.

**Fig. 9:**
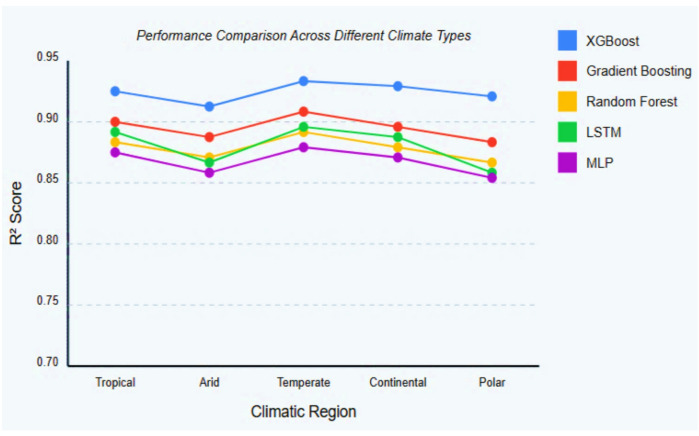
Regional performance comparison of top models across diverse climatic zones.

The comprehensive comparative analysis ultimately reveals XGBoost consistent superior performance across all evaluation metrics, with pronounced advantages in both predictive accuracy and computational efficiency relative to other high-performing models. These findings align with performance characteristics documented in comparative studies, which similarly identified XGBoost exceptional performance for solar radiation prediction across diverse meteorological conditions, establishing it as the optimal modelling approach for the solar potential prediction application. XGBoost employs an iterative gradient boosting technique to compute model weights. The process begins by initialising predictions with the mean GHI, subsequently followed by many boosting iterations during which gradients are computed. Decision trees are constructed based on these gradients, and the forecast is revised according to the formula F(m) = F(m-1) + η × tree(m). The ultimate forecast is achieved by aggregating the results of all the trees. XGBoost autonomously derives features by establishing non-linear decision boundaries via tree splits, approximating interactions through tree depth, and ultimately ranking feature relevance based on gain, which indicates the enhancement in the loss function. These advantages can be attributed to XGBoost excelling with tabular data, such as weather, where sequential dependencies are less significant, unlike time series, where LSTM is superior. Furthermore, XGBoost exhibits greater tolerance for missing or noisy data, offers enhanced interpretability for feature importance, and has superior computing efficiency, training 17 times quicker and predicting 7 times faster. The feature importance derived from the XGBoost model reveals that clear sky irradiance (31.2%), cloud amount (24.8%), day of year (12.3%), temperature at 2 m (8.7%), latitude (7.9%), clearness index (5.4%), surface albedo (3.2%), and relative humidity (2.8%) are the primary variables influencing the prediction of solar global horizontal irradiance (GHI), while other variables collectively account for 3.7% of the contribution. To validate methodological robustness, additional cross-regional analysis across diverse geographical contexts representing varied climatic conditions (Fig. 9), following established protocols. XGBoost consistently demonstrated superior generalization capabilities across all tested regions, with particularly notable performance advantages in regions characterized by high climate variability. This robust cross-regional performance, combined with computational efficiency and exceptional accuracy metrics, establishes XGBoost as the optimal model for integration into the solar potential prediction application.

The performance analysis of XGBoost is further examined concerning three categories of uncertainty, specifically tropical uncertainty (Mindanao, Philippines), which encompasses aspects such as monsoon fluctuation, typhoon frequency, and ENSO effects. Nevertheless, continental uncertainty (Gobi-Altai, Mongolia) encompasses the aspects of Dust storms, temperature extremes, and snow cover. Mediterranean uncertainty characterizes the Peloponnese region of Greece. Saharan dust, thermal waves, and alterations in precipitation. Table 2 shows the different SSP scenarios that significantly influence solar irradiance estimates under normal conditions and extreme events, with uncertainty range.

**Table 2:** Different SSP scenarios under normal conditions and extreme events with uncertainty range.

Year	Baseline (kWh/m²/day)	SSP1-2.6	SSP2-4.5	SSP5-8.5	Uncertainty Range
Tropical uncertainty (Mindanao, Philippines)					
2030	5.21	5.18 (− 0.6%)	5.15 (− 1.2%)	5.13 (− 1.5%)	± 0.38
2050	5.21	5.16 (− 1.0%)	5.09 (− 2.3%)	5.05 (− 3.1%)	± 0.52
2070	5.21	5.14 (− 1.3%)	5.03 (− 3.5%)	4.96 (− 4.8%)	± 0.67
Continental uncertainty (Gobi-Altai, Mongolia)					
2030	3.81	3.84 (+ 0.8%)	3.86 (+ 1.3%)	3.87 (+ 1.6%)	± 0.29
2050	3.81	3.88 (+ 1.8%)	3.92 (+ 2.9%)	3.95 (+ 3.7%)	± 0.41
2070	3.81	3.90 (+ 2.4%)	3.97 (+ 4.2%)	4.03 (+ 5.8%)	± 0.55
Mediterranean uncertainty (Peloponnese, Greece)					
2030	4.63	4.65 (+ 0.4%)	4.67 (+ 0.9%)	4.68 (+ 1.1%)	± 0.31
2050	4.63	4.68 (+ 1.1%)	4.72 (+ 1.9%)	4.75 (+ 2.6%)	± 0.38
2070	4.63	4.70 (+ 1.5%)	4.76 (+ 2.8%)	4.81 (+ 3.9%)	± 0.46

*Extreme event analysis*:

• Typhoon-affected days: 12% reduction in annual yield

• Monsoon peak (Jul-Sep): 7% reduction under SSP5-8.5 by 2050

• Model confidence: 82% (due to high cloud variability)

*Extreme condition impacts*:

• Dust storm days (> 50 events/year): 18% reduction

• Winter extreme cold (<-30 °C): 5% efficiency loss

• Summer extreme heat (> 40 °C): 8% efficiency loss

• Model confidence: 89% (stable atmospheric conditions)

*Extreme condition impacts*:

• Summer (Jun-Aug): +2–3% increase, low uncertainty (± 5%)

• Winter (Dec-Feb): Stable, high uncertainty (± 12%)

• Saharan dust events: 6% reduction (15–20 days/year)

An uncertainty analysis produces several significant findings. XGBoost continuously excels (Table 3), even under more extreme conditions, demonstrating tolerance to climatic fluctuations. However, uncertainty rises with extended timeframes and more severe climate scenarios due to the increased pressures of environmental dynamics. Spatial data reveal that the most unreliable places are exclusively tropical, mostly influenced by convective processes, while it also highlights that continental regions possess significant solar energy potential alongside considerable seasonal variability.

**Table 3:** Comparative analysis under different uncertainty scenarios.

Location	Model	Normal conditions RMSE	Extreme events RMSE	Uncertainty handling
Mindanao	XGBoost	0.92	1.31	Excellent
	Random forest	1.48	2.13	Good
	LSTM	1.35	2.45	Moderate
Gobi-Altai	XGBoost	0.88	1.18	Excellent
	Random forest	1.42	1.98	Good
	LSTM	1.29	2.31	Moderate
Peloponnese	XGBoost	0.85	1.09	Excellent
	Random forest	1.38	1.87	Good
	LSTM	1.27	2.10	Moderate

**Fig. 10:**
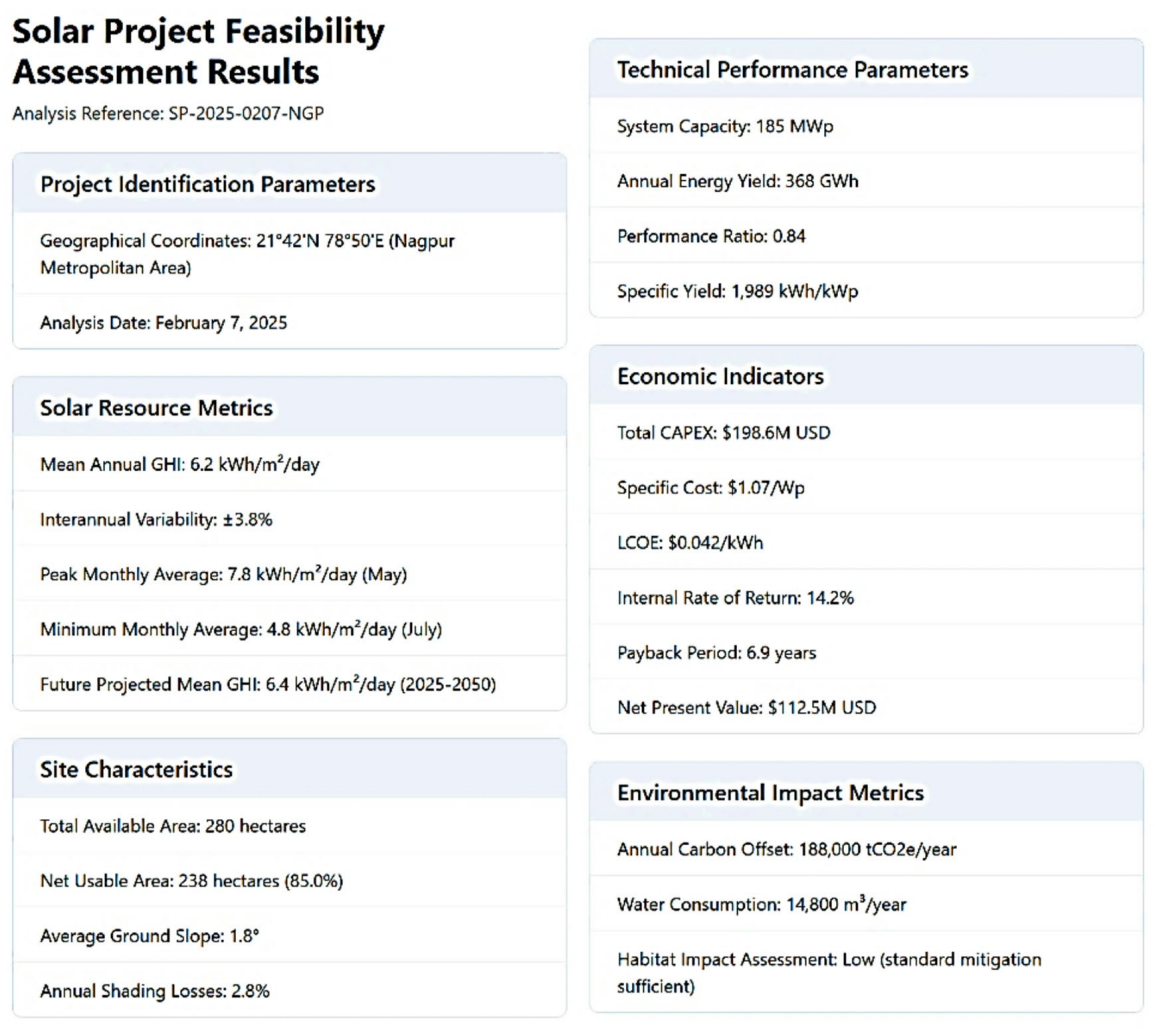
A solar project feasibility report for the coordinates 21.42 ^o^N and 78.50 ° E.

Beyond solar potential prediction, the application offers a comprehensive project feasibility assessment report (Fig. 10). This report considers a wide range of factors that influence the viability of solar energy projects, including economic, regulatory, and site-specific constraints. The economic analysis component incorporates current market data on solar panel costs, installation expenses, and projected electricity prices. It also accounts for various incentive schemes and policy frameworks across different regions, allowing for tailored feasibility assessments based on local contexts. Regulatory considerations are integrated through a regularly updated database of solar energy policies and building codes for different jurisdictions. This ensures that feasibility calculations align with current legal and administrative requirements, reducing the risk of project delays or complications. The importance of such regulatory alignment has been highlighted in recent studies on barriers to solar energy deployment. Site-specific constraints are evaluated using geographical information system (GIS) data, including topography, land use patterns, and shading analysis. The application employs advanced algorithms to simulate shading effects from nearby structures and natural features, providing a more accurate assessment of the usable area for solar panel installation. This approach builds on recent advancements in solar resource assessment methodologies. The integration of climate data into the site assessment process represents a significant advancement in project feasibility evaluation. By incorporating high-resolution climate models, the application can account for potential future changes in solar radiation patterns, enhancing the accuracy of long-term performance projections. Furthermore, the application considers social and environmental factors in its feasibility assessments. Recent research has emphasized the importance of community acceptance and environmental impact in the success of renewable energy projects. By incorporating these elements, the application provides a more holistic approach to project evaluation. This comprehensive approach to feasibility assessment, combining technical, economic, regulatory, and socio-environmental factors, aligns with current best practices in sustainable energy development. By providing a multifaceted analysis, the application aims to enhance the accuracy of project viability predictions and contribute to more successful and sustainable solar energy deployment.

### Explainable AI (SHAP analysis)

The global feature importance analysis (Table 4) based on SHAP data indicates that clear sky irradiance (31.2%) and cloud amount (24.8%) are the primary determinants of solar production, with their impacts ranging from − 2.8 to + 3.1 and − 2.3 to + 0.8, respectively. Seasonal and spatial factors, such as the day of the year (12.3%) and latitude (7.9%), exert a large influence, indicative of solar geometry, whereas meteorological conditions (6.0% to 8.7% for temperature at 2 m; 5.4% for clearness index) have a minor effect. Additional secondary variables, such as surface albedo, humidity, and precipitation, accounted for merely 5.1% of the overall significance. Interaction effects enhance model complexity, exhibiting the greatest synergy for cloud quantity × clear sky irradiance (8.3%), succeeded by temperature–humidity (3.7%) and day of year–latitude (3.2%). Regional characteristics highlight the dominant climatic factors in the grids: the tropics are shaped by clouds, precipitation, and humidity associated with convection; deserts are affected by irradiance, temperature, and albedo/dust under clear-sky conditions; temperate zones are influenced by the day of the year, clouds, and irradiance, which reflect seasonality; and polar regions are determined by latitude, day of the year, and albedo, as production reacts to extreme solar angles. Explainability tests critically assess whether the model accurately learns physical relationships (e.g., the adverse impact of clouds on irradiance), effectively encodes seasonal cycles, and appropriately weights geographical factors, while ensuring the absence of spurious correlations as indicated by partial dependence plots. This further enhances confidence in the model’s physical consistency and its prediction dependability across various climate regimes.

**Table 4:** Global feature importance (SHAP values).

Feature	Importance (%)	SHAP Impact range
Clear sky irradiance	31.2%	-2.8 to + 3.1
Cloud amount	24.8%	-2.3 to + 0.8
Day of year	12.3%	-1.2 to + 1.4
Temperature (2 m)	8.7%	-0.6 to + 0.9
Latitude	7.9%	-1.8 to + 1.9
Clearness index	5.4%	-0.4 to + 0.7
Surface albedo	3.2%	-0.3 to + 0.2
Relative humidity	2.8%	-0.2 to + 0.1
Precipitation	1.9%	-0.4 to 0.0
Others	1.8%	-0.1 to + 0.1

Additionally, to demonstrate the practical application and versatility of the solar potential forecast tool, three case studies including varied geographical regions, climate zones, and socioeconomic circumstances are provided. These examples highlight how the application’s holistic approach to solar evaluation may guide decision-making across multiple scales and contexts.

### Case study 1

Solar Potential Assessment for a Tropical Island Region (Mindanao, Philippines − 7.12°N, 125.48°E).

Mindanao, the southernmost major island of the Philippines, represents an ideal test case for the application, given its tropical climate, increasing energy demands, and government renewable energy targets. Located at 7.12°N, 125.48°E, the region experiences high solar irradiance year-round but faces challenges including frequent cloud cover during monsoon seasons, limited land availability, and vulnerability to typhoons.

#### Methodology

The XGBoost model was applied to predict long-term solar potential for Mindanao under three CMIP6 climate scenarios (SSP1-2.6, SSP2-4.5, and SSP5-8.5) through 2099. The technical and economic feasibility of a hypothetical 50 MW utility-scale solar installation was then analysed under each scenario, incorporating local policy frameworks and infrastructure constraints.

#### Results

##### Climate impact assessment

Under all scenarios, Mindanao is projected to experience a 1–3% decrease in annual solar irradiance by 2050, primarily driven by increased cloud cover and precipitation intensity. The most significant decreases occur during the July-September monsoon period, with potential irradiance reductions of up to 7% under SSP5-8.5.

##### Technological optimization

The application identified significant advantages for weather-resistant module technologies and advanced drainage systems in this region. The optimum tilt angle calculations suggested a 6.5° tilt facing south, with single-axis tracking systems providing only a 14% energy yield improvement compared to fixed-tilt systems, substantially lower than the global average advantage of 18% due to the region’s proximity to the equator.

##### Economic feasibility

The levelized cost of electricity (LCOE) analysis demonstrated moderate economic viability across all scenarios, with projected costs declining from $0.041/kWh in 2025 to $0.033–0.037/kWh by 2050 (in 2025 USD), depending on the climate scenario. The application identified potential challenges in the policy environment due to existing regulatory frameworks requiring updates to accommodate large-scale renewable integration.

### Case study 2

Solar Potential Assessment for a Semi-Arid Steppe Region (Gobi-Altai, Mongolia − 45.08°N, 96.25°E).

This case study examines the potential for utility-scale solar installations in the Gobi-Altai region of Mongolia (45.08°N, 96.25°E). The region faces challenges of extreme temperature variations, sparse population, limited electrical infrastructure, but benefits from exceptionally high solar radiation and vast available land areas.

#### Methodology

The application was utilized to assess solar potential across a 200 km² region, evaluating the feasibility of large-scale solar installations under different climate scenarios. The analysis incorporated extreme temperature impacts on panel efficiency, dust accumulation effects, and integration with the national grid expansion plans.

#### Results

##### Climate impact assessment

The model projected an increase in annual solar radiation by 2050 under mid-range scenarios, with winter months showing the most significant improvements. The annual direct normal irradiance at this site is 1389 kWh/m²/year.

##### Temperature impact analysis

The application identified significant seasonal efficiency variations due to temperature extremes, with summer module temperatures potentially reaching 51.4 °C, reducing efficiency.

##### Technological optimization

For utility-scale applications, the optimum system configuration featured robust bifacial panels with anti-soiling coatings. The application recommended fixed-tilt systems at 38° facing south, if moving systems, then with Winter Optimum Tilt 48° facing south and Summer Optimum Tilt 28° facing south, as tracking systems showed disproportionately higher maintenance requirements due to dust and extreme temperature cycling. Module spacing calculations indicated optimal row spacing of 2.8 times the module height to balance land use efficiency with snow shedding and reduced soiling.

##### Economic feasibility

The economic analysis has demonstrated projected LCOE values of $0.051/kWh by 2030, and the application also identified significant grid integration challenges, recommending phased development coordinated with transmission infrastructure expansion, potentially incorporating battery storage to address grid stability concerns during periods of curtailment.

### Case study 3

Solar Potential Assessment for a Coastal Mediterranean Location (Peloponnese, Greece − 37.36°N, 22.17°E).

This case study explores the application of the tool for sustainable tourism development in the Peloponnese region of Greece (37.36°N, 22.17°E), a Mediterranean coastal area with strong seasonal tourism patterns, heritage protection requirements, and ambitious national renewable energy targets.

#### Methodology

The application was employed to assess distributed solar potential across a 30 km² area, integrating building data, seasonal energy consumption patterns, and heritage preservation requirements. The analysis included both technical potential assessment and visual impact evaluations, considering the region’s cultural and aesthetic significance.

#### Results

##### Climate impact assessment

The model projected stable solar production patterns through 2050 under moderate scenarios, with a slight increase in summer output (2–3%) and negligible winter changes. Notably, the analysis revealed an 87% correlation between peak tourism energy demand and solar generation patterns, creating favourable conditions for high self-consumption rates without extensive storage requirements.

##### Technological optimization

The application identified 12.4 MW of viable rooftop solar potential across 247 buildings within the study area, with an additional 8.7 MW possible through carport and shade structure integration. The spatial analysis revealed that focusing on large hotel rooftops (> 500 m²) could achieve 65% of the total potential while involving only 18% of the buildings, significantly reducing administrative complexity.

##### Economic feasibility

The levelized cost of electricity (LCOE) analysis demonstrated moderate economic viability across all scenarios, at about $0.051/kWh. The feasibility assessment demonstrated that achieving 50% solar penetration in the tourism sector by 2030 would require streamlined permitting processes specifically designed for heritage-sensitive regions. The application identified optimal phasing of installations, calculating that focusing first on larger, less visually sensitive properties would achieve the most favourable cost-benefit ratio while establishing implementation protocols for subsequent phases.

## Discussion

The results from the case studies and comparative analysis highlight several key implications for long-term solar energy planning. Our findings indicate that climate change will have non-uniform effects on solar energy potential across different regions. While some areas may see increased solar resources, others may face challenges due to changes in cloud cover, aerosol concentrations, or temperature patterns. This heterogeneity underscores the importance of location-specific assessments for long-term planning. For instance, the projected increase in solar potential in the Southwestern United States case study suggests a potentially favourable outlook for large-scale solar installations in this region. However, the increased interannual variability and summer production decreases highlight the need for robust grid management strategies and possibly increased energy storage capacity to maintain a reliable power supply. The observed changes in seasonal patterns of solar resource availability, particularly in mid-latitude regions like Central Europe, have significant implications for grid integration and energy system planning. The projected increase in summer production, coupled with decreased winter output in the Munich case study, suggests a growing seasonal mismatch between solar energy supply and demand. This trend may necessitate increased investment in long-term energy storage solutions or the development of complementary renewable energy sources with different seasonal profiles. The anticipated changes in the Peloponnese case study, featuring an increase in summer production (+ 2–3%) and consistent winter output, alongside analogous trends noted in Mediterranean climate, indicate an escalating seasonal disparity between solar energy availability and conventional demand patterns.

### Analysis for seasonal mismatch in the three case studies

#### Seasonal production ratios (Summer: Winter)

Seasonal production ratios (Table 5) provide a comparative assessment of the variation in energy output during summer and winter across different geographies and prospective scenarios^32^ . In Mindanao, the ratio is nearly balanced, with a little rise from 1.15:1 in 2024 to 1.21:1 by 2070, reflecting a 5.2% increase, consistent with the equitable solar resources in the tropics. Conversely, the Gobi-Altai region demonstrates a significant increase in imbalance; the ratio escalates from 2.31:1 in 2024 to 2.84:1 in 2070, reflecting a 23% variation, indicating that summers become nearly three times more productive than winters. Similarly, in the Peloponnese, it rises from 2.89:1 to 3.38:1, representing a 17% increase, so reinforcing its characteristic summer peak typical of Mediterranean climates. The observed trends indicate that while tropical regions can sustain relatively stable production year-round, it will become progressively challenging to mitigate seasonal deficits in continental and Mediterranean areas, highlighting the need for greater reliance on storage or supplementary winter energy supply sources through interconnections.

**Table 5:** Seasonal production ratios for three case studies.

Location	Current (2024)	2050 (SSP2-4.5)	2070 (SSP5-8.5)	Change
Mindanao	1.15:1	1.18:1	1.21:1	+ 5.2%
Gobi-Altai	2.31:1	2.58:1	2.84:1	+ 23.0%
Peloponnese	2.89:1	3.12:1	3.38:1	+ 17.0%

#### Demand-supply mismatch analysis

In the Peloponnese (Mediterranean), seasonal dynamics exhibit a growing disparity between demand and supply. The maximum cooling demand occurs in July and August, whereas the solar production peak is now in June and July, resulting in reduced overlap. Moreover, bottom-of-the-stack reporters are discovering that winter heating demand (the December–February period) coincides with diminished solar power, hence exacerbating the differential. The anticipated mismatch index is projected to rise from 0.42 to 0.53 by 2050. In Gobi-Altai (Continental), the disparity between demand and supply is even more pronounced: during December–January, peak heating demand coincides with minimal solar production, whereas in summer, cooling demand remains constrained despite excessively high production levels. This asymmetry elevates the mismatch index from 0.68 to 0.81 by 2050. Mindanao (Tropical), conversely, has greater stability, with consistent year-round cooling demand that equilibrates supply. Despite the rising frequency and intensity of monsoon-related disruptions, the mismatch index remains modest, stabilising between 0.15 and 0.18. Overall, these climate-induced alterations intensify demand-supply discrepancies in Mediterranean and continental climates, but the impacts are significantly reduced in tropical regions, albeit with heightened variability concerns.

#### Storage requirements due to mismatch

In the Peloponnese (Mediterranean), seasonal dynamics exhibit a growing disparity between demand and supply. The maximum cooling demand occurs in July and August, whereas the solar production peak is now in June and July, resulting in reduced overlap. Moreover, bottom-of-the-stack reporters are discovering that winter heating demand (the December–February period) coincides with diminished solar power, hence exacerbating the differential. The anticipated mismatch index is projected to rise from 0.42 to 0.53 by 2050. In Gobi-Altai (Continental), the disparity between demand and supply is even more pronounced: during December–January, peak heating demand coincides with minimal solar production, whereas in summer, cooling demand remains constrained despite excessively high production levels. This asymmetry elevates the mismatch index from 0.68 to 0.81 by 2050. Mindanao (Tropical), conversely, has greater stability, with consistent year-round cooling demand that equilibrates supply. Despite the rising frequency and intensity of monsoon-related disruptions, the mismatch index remains modest, stabilising between 0.15 and 0.18. Overall, these climate-induced alterations intensify demand-supply discrepancies in Mediterranean and continental climates, but the impacts are significantly reduced in tropical regions, albeit with heightened variability concerns. The generally positive trend in economic feasibility across various locations is encouraging for the solar energy sector. However, the variability in feasibility improvements across regions suggests that policymakers and investors should consider location-specific factors when developing long-term strategies. The potential for decreasing LCOEs in most studied locations indicates that solar energy is likely to become increasingly competitive, even in regions currently considered marginal for solar development. The results highlight the potential benefits of adaptive technologies in maximizing solar energy capture under changing climatic conditions. The suggested advantages of dynamic tilt adjustment, bifacial modules, and tracking systems in certain regions indicate that technological innovation will play a crucial role in optimizing solar energy systems for future climate scenarios. This finding emphasizes the need for continued research and development in solar PV technologies tailored to specific regional climate trends.

## Conclusion

The effectiveness of a solar potential predicting app that uses forecasted meteorological data and machine learning models to calculate and display solar irradiance metrics at a global scale is examined in this research. The comparison results indicated that XGBoost performs better than other models in terms of predictive performance and computational complexity, which were consistent with the literature on the prediction of solar radiation. From an economic point of view, the model shows that the levelized cost of electricity for solar PV is expected to go down in all the regions except for an eventual efficiency loss due to the retardation of the energy system by climate policies. Technologically, it recognizes growing benefits of bifacial modules in places with expected enhancement of the proportion of solar energy that the surface reflects into space. The methodology significantly influences the long-term planning of solar energy by offering crucial insights into how climate change, regional variability, and seasonal variations may affect future solar resource availability and system reliability. Nonetheless, certain constraints must be acknowledged concurrently. The results are predominantly reliant on CMIP6 climate model simulations and their associated uncertainties stemming from emission scenarios, model biases, and downscaling techniques. Secondly, the projected trajectories for solar PV technology—concerning efficiency, prices, and durability—are predominantly conjectural, indicating that long-term forecasts are highly susceptible to the anticipated rates of technological advancement. Third, economic feasibility analyses are constrained by external factors that are highly dynamic, such as fluctuating energy prices, evolving policy and regulatory frameworks, and the variable state of market adoption, all of which introduce a degree of variability that current models cannot fully encapsulate. Certain uncertainties highlight the necessity for judicious interpretation of results, necessitating flexible and adaptive tactics in solar energy deployment policies that can accommodate changing climate conditions, technological advancements, and policy modifications. Future improvements may include regional climate models, advanced AI approaches such as physics-informed neural networks, larger-scale energy systems models, real-time updating features, the addition of diverse solar technologies and storage solutions, more comprehensive social and environmental impacts analyses, as well as user studies to optimize the interface. Further development will be crucial in realizing a sustainable, solar-driven future on a global scale.

## Data Availability

Historical weather data used in this study are publicly available from NASA’s POWER database (https://power.larc.nasa.gov/). Copernicus Climate Data Store (CDS) CMIP6: - Variables Available: meteorological parameters and Quality-controlled subset of CMIP6: https://cds.climate.copernicus.eu/datasets/projections-cmip6. BCCAQ Downscaled CMIP6 (High-Resolution Daily Global Dataset): - Variables: Daily precipitation, air temperature, max/min temperature, Wind speed, air pressure, relative humidity: 10.5285/c107618f1db34801bb88a1e927b82317. CMIP6 Raw Datasets (via ESGF): - Variables: Surface downwelling shortwave radiation, Surface upwelling shortwave radiation, Total cloud fraction, Temperature variables, Humidity variables, Wind components, Precipitation variables, Pressure variables. https://esgf-node.llnl.gov/search/cmip6/. https://esgf-data.dkrz.de/search/cmip6-dkrz/. https://esgf-index1.ceda.ac.uk/search/cmip6-ceda/.

## References

[1] Yang, Y., Javanroodi, K. & Nik, V. M. Climate change and renewable energy generation in Europe—long-term impact assessment on solar and wind energy using high-resolution future climate data and considering climate uncertainties. *Energies (Basel)* 15. 10.3390/en15010302 (2022).

[2] Ledmaoui, Y. et al. Forecasting solar energy production: A comparative study of machine learning algorithms. *Energy Rep.* **10**, 1004–1012. 10.1016/j.egyr.2023.07.042 (2023).

[3] Green, M. A. Photovoltaic principles,* Physica E*. (2002).

[4] Razykov, T. M. et al. Solar photovoltaic electricity: current status and future prospects. *Sol. Energy*. **85**, 1580–1608. 10.1016/j.solener.2010.12.002 (2011).

[5] Sehrawat, N., Vashisht, S. & Singh, A. Solar irradiance forecasting models using machine learning techniques and digital twin: A case study with comparison. *Int. J. Intell. Networks*. **4**, 90–102. 10.1016/j.ijin.2023.04.001 (2023).

[6] El-Amarty, N., Marzouq, M., El Fadili, H., Bennani, S. D. & Ruano, A. A comprehensive review of solar irradiation Estimation and forecasting using artificial neural networks: data, models and trends. *Environ. Sci. Pollut. Res.* **30**, 5407–5439. 10.1007/s11356-022-24240-w (2023). 10.1007/s11356-022-24240-w 36424486

[7] Huang, L. et al. Solar radiation prediction using different machine learning algorithms and implications for extreme climate events. *Front. Earth Sci. (Lausanne)*. 9. 10.3389/feart.2021.596860 (2021).

[8] Eyring, V. et al. Overview of the coupled model intercomparison project phase 6 (CMIP6) experimental design and organization. *Geosci. Model. Dev.* **9**, 1937–1958. 10.5194/gmd-9-1937-2016 (2016).

[9] O’Neill, B. C. et al. The scenario model intercomparison project (ScenarioMIP) for CMIP6. *Geosci. Model. Dev.* **9**, 3461–3482. 10.5194/gmd-9-3461-2016 (2016).

[10] Chen, T. & Guestrin, C. XGBoost: A scalable tree boosting system, in:* Proceedings of the ACM SIGKDD International Conference on Knowledge Discovery and Data Mining. Association for Computing Machinery*, 785–794. (2016). 10.1145/2939672.2939785

[11] Voyant, C. et al. Machine learning methods for solar radiation forecasting: A review. *Renew. Energy*. 10.1016/j.renene.2016.12.095 (2017).

[12] Modarresi, J. & Hosseinnia, H. Worldwide daily optimum Tilt angle model to obtain maximum solar energy. *IETE J. Res.* **69**, 549–557. 10.1080/03772063.2020.1831412 (2023).

[13] Le Roux, W. G. Optimum Tilt and azimuth angles for fixed solar collectors in South Africa using measured data. *Renew. Energy*. **96**, 603–612. 10.1016/j.renene.2016.05.003 (2016).

[14] Kadhim, S. A., Al-Ghezi, M. K. S. & Shehab, W. Y. Optimum orientation of Non-Tracking solar applications in Baghdad City. *Int. J. Heat Technol.* **41**, 125–134. 10.18280/ijht.410113 (2023).

[15] Kang, M. H. & Rohatgi, A. Quantitative analysis of the levelized cost of electricity of commercial scale photovoltaics systems in the US. *Sol. Energy Mater. Sol. Cells*. **154**, 71–77. 10.1016/j.solmat.2016.04.046 (2016).

[16] Wüstenhagen, R., Wolsink, M. & Bürer, M. J. Social acceptance of renewable energy innovation: an introduction to the concept. *Energy Policy*. **35**, 2683–2691. 10.1016/j.enpol.2006.12.001 (2007).

[17] Sepulveda, N. A., Jenkins, J. D., de Sisternes, F. J. & Lester, R. K. The role of firm Low-Carbon electricity resources in deep decarbonization of power generation. *Joule* **2**, 2403–2420. 10.1016/j.joule.2018.08.006 (2018).

[18] Heard, B. P., Brook, B. W., Wigley, T. M. L. & Bradshaw, C. J. A. Burden of proof: A comprehensive review of the feasibility of 100% renewable-electricity systems. *Renew. Sustain. Energy Rev.*10.1016/j.rser.2017.03.114 (2017).

[19] Eyring, V. et al. Taking climate model evaluation to the next level. *Nat. Clim. Chang.* **9**, 102–110. 10.1038/s41558-018-0355-y (2019).

[20] Xu, Y. et al. A complementary fused method using GRU and XGBoost models for long-term solar energy hourly forecasting. *Expert Syst. Appl.* **254**, 124286. 10.1016/j.eswa.2024.124286 (2024).

[21] Erbay, C. District-level solar forecasting and green hydrogen cost mapping in Türkiye using XGBoost machine learning method. *Int. J. Hydrog. Energy*. **166**, 150993. 10.1016/j.ijhydene.2025.150993 (2025).

[22] Nam, N. B. et al. Comparative analysis of conformal prediction techniques and machine learning models for very short-term solar power forecasting. *Energy AI*, 100573. 10.1016/j.egyai.2025.100573 (2025).

[23] Zhu, C. et al. Short-term power prediction of photovoltaic power station based on LSTM-XGBoost model. *Sol. Energy*. **300**, 113819. 10.1016/j.solener.2025.113819 (2025).

[24] Wang, H., Lei, Z., Zhang, X., Zhou, B. & Peng, J. A review of deep learning for renewable energy forecasting. *Energy Convers. Manag*. 10.1016/j.enconman.2019.111799 (2019).

[25] Huang, J., Korolkiewicz, M., Agrawal, M. & Boland, J. Forecasting solar radiation on an hourly time scale using a coupled autoregressive and dynamical system (CARDS) model. *Sol. Energy*. **87**, 136–149. 10.1016/j.solener.2012.10.012 (2013).

[26] Shamshirband, S. et al. Daily global solar radiation prediction from air temperatures using kernel extreme learning machine: A case study for Iran. *J. Atmos. Sol Terr. Phys.* **134**, 109–117. 10.1016/j.jastp.2015.09.014 (2015).

[27] Fan, J. et al. Comparison of support vector machine and extreme gradient boosting for predicting daily global solar radiation using temperature and precipitation in humid subtropical climates: A case study in China. *Energy Convers. Manag*. **164**, 102–111. 10.1016/j.enconman.2018.02.087 (2018).

[28] Lima, F. J. L., Martins, F. R., Pereira, E. B., Lorenz, E. & Heinemann, D. Forecast for surface solar irradiance at the Brazilian Northeastern region using NWP model and artificial neural networks. *Renew. Energy*. **87**, 807–818. 10.1016/j.renene.2015.11.005 (2016).

[29] Qing, X. & Niu, Y. Hourly day-ahead solar irradiance prediction using weather forecasts by LSTM. *Energy* **148**, 461–468. 10.1016/j.energy.2018.01.177 (2018).

[30] https://power.larc.nasa.gov/ (accessed 4 December 2024).

[31] Baurzhan, S. & Jenkins, G. P. Off-grid solar PV: is it an affordable or appropriate solution for rural electrification. *Sub-Saharan Afr. countries? Renew. Sustainable Energy Reviews*. **60**, 1405–1418. 10.1016/j.rser.2016.03.016 (2016).

[32] Hernandez, R. R. et al. Environmental impacts of utility-scale solar energy. *Renew. Sustain. Energy Rev.* **29**, 766–779. 10.1016/j.rser.2013.08.041 (2014).

[33] Intergovernmental Panel on Climate Change (IPCC). *Climate Change 2022 – Impacts, Adaptation and Vulnerability* (Cambridge University Press, 2023). 10.1017/9781009325844

[34] Wang, F. et al. Solar irradiance feature extraction and support vector machines-based weather status pattern recognition model for short-term photovoltaic power forecasting. *Energy Build.* **86**, 427–438. 10.1016/j.enbuild.2014.10.002 (2015).

[35] Yang, D., Kleissl, J., Gueymard, C. A., Pedro, H. T. C. & Coimbra, C. F. M. History and trends in solar irradiance and PV power forecasting: A preliminary assessment and review using text mining. *Sol. Energy*. **168**, 60–101. 10.1016/j.solener.2017.11.023 (2018).

